# LC-MS/MS-Based Site-Specific N-Glycosylation Analysis of VEGFR-IgG Fusion Protein for Sialylation Assessment Across IEF Fractions

**DOI:** 10.3390/molecules29225393

**Published:** 2024-11-15

**Authors:** Kwang Hoe Kim, Eun Sun Ji, Ju Yeon Lee, Ju Hwan Song, Yeong Hee Ahn

**Affiliations:** 1 CellKey Inc., Cheongju 28160, Republic of Korea; khkim@cellkey.co.kr (K.H.K.); eunsunji@cellkey.co.kr (E.S.J.); 2 Digital Omics Research Center, Korea Basic Science Institute, Ochang 28119, Republic of Korea; jylee@kbsi.re.kr (J.Y.L.); sjhs5904@kbsi.re.kr (J.H.S.); 3 Department of Bio-Analytical Science, University of Science and Technology, Daejeon 34113, Republic of Korea; 4 Department of Biotechnology, Graduate School, Korea University, Seoul 02841, Republic of Korea; 5 Department of Biomedical Science, Cheongju University, Cheongju 28160, Republic of Korea

**Keywords:** Eylea fusion protein, sialylated N-glycopeptide, mass spectrometry

## Abstract

The glycosylation profile of therapeutic proteins significantly influences their efficacy, stability, and immunogenicity. Sialylation is crucial for the biological activity and pharmacokinetics of fusion proteins used in treating angiogenic disorders, making sialic acid levels a critical quality attribute in the development and production of biologics. In this study, we employed a mass spectrometry-based approach to assess sialylation levels through site-specific N-glycosylation analysis. To validate the method’s effectiveness, IEF fractions (acidic, main, and basic) obtained from the production media of the VEGFR-IgG fusion protein and anticipated to exhibit varying sialylation levels were analyzed. Our analytical method successfully evaluated the sialylation levels of each domain—IgG, VEGFR-1, and VEGFR-2—within the Fc-fusion protein. The results confirm that the overall sialylation level of the Fc-fusion protein correlated with the levels observed across the IEF fractions. This finding highlights the value of LC-MS/MS-based sialylation monitoring as a crucial tool for biosimilar development and quality control, particularly in optimizing target protein production. Additionally, glycopeptide-based LC-MS analysis enables site-specific sialylation evaluation, ensuring consistent profiles for robust quality assurance.

## 1. Introduction

The precise characterization of recombinant fusion glycoproteins is of paramount importance in the biopharmaceutical industry, as glycosylation profoundly influences the stability, efficacy, and immunogenicity of therapeutic proteins [1,2,3]. A recombinant fusion protein used to treat various angiogenic disorders exemplifies the complexity and significance of glycosylation patterns, particularly sialylation [4,5,6]. Sialylation, the addition of sialic acid residues to glycoproteins, plays a crucial role in determining the biological activity, half-life, and overall therapeutic performance of biopharmaceuticals [7,8].

Understanding the sialylation of fusion glycoproteins is crucial as it can influence a drug’s therapeutic efficacy and pharmacokinetics [9,10]. Variations in glycosylation can lead to differences in the drug’s interaction with cell surface receptors, its clearance from the bloodstream, and its overall bioactivity [11,12,13]. Therefore, this research not only contributes to the quality control and consistency of fusion glycoprotein production but also enhances our understanding of how glycosylation affects the function of fusion proteins used in medical treatments [9,14,15].

Liquid chromatography–mass spectrometry (LC-MS) has emerged as a powerful analytical tool for the detailed analysis of glycoproteins, offering high sensitivity and specificity for the identification and quantification of glycopeptides [16,17,18]. LC-MS/MS offers superior specificity by leveraging mass-to-charge (*m*/*z*) ratios to precisely monitor sialylation levels. Moreover, compared with capillary electrophoresis, LC-MS/MS facilitates faster data acquisition and simplifies sample handling, enhancing the reliability of repeated analyses. Consequently, this approach enables more accurate and efficient sialylation analysis compared with conventional techniques [19].

The importance of sialylation in therapeutic proteins cannot be overstated. Variations in sialylation can affect the protein’s interaction with cellular receptors, its clearance from the bloodstream, and its immunogenicity [20,21]. Therefore, an accurate assessment of sialylation levels is critical for ensuring the consistency and quality of fusion glycoprotein production, as well as for optimizing its therapeutic efficacy [22,23].

Aflibercept, marketed under the brand name Eylea, is a recombinant fusion protein designed for therapeutic use. It is formulated as a sterile, clear, and colorless–pale yellow aqueous solution intended for intravitreal injection. Aflibercept consists of a fusion protein combining parts of VEGFR-1 and VEGFR-2 linked to the Fc region of human IgG1. The excipients include polysorbate 20, sodium chloride, monobasic and dibasic sodium phosphate, and water for injection, with the pH adjusted for stability. VEGFRs are a family of tyrosine kinase receptors that play a pivotal role in mediating the effects of vascular endothelial growth factors (VEGFs), which are critical for blood vessel formation (angiogenesis) [24]. Aflibercept exhibits glycan diversity, particularly in its VEGFR domains, with notable differences in sialylation levels. These variations in sialylation can influence aflibercept’s ability to bind to VEGF165, where increased sialylation may occasionally result in reduced binding affinity. Despite these differences, comparisons across multiple batches of aflibercept demonstrate that its overall therapeutic efficacy remains stable across different formulations [25,26].

In this study, we present a comparative analysis of the sialylated N-glycopeptide levels in a VEGFR-IgG fusion protein using LC-MS/MS techniques. Aflibercept, a biotherapeutic VEGFR-IgG fusion protein, consists of a homodimer of vascular endothelial growth factor receptors 1 and 2 (VEGFR-1 and VEGFR-2) fused to the Fc portion of human IgG and contains five known N-linked glycosylation sites: two in the VEGFR-1 region, two in the VEGFR-2 region, and one in the IgG Fc region. IEF (isoelectric focusing) fractionation, an electrophoresis technique that separates proteins based on their isoelectric point (pI), was used to purify the aflibercept fractions, each containing protein molecules with various charge variants primarily influenced by post-translational modifications, particularly glycosylation. Eylea is formulated to include aflibercept molecules with pI values between pH 6.5 and pH 7.5. For Eylea biosimilar production, both upstream and downstream processes must be optimized to ensure the biosimilar’s aflibercept molecules have physicochemical properties closely matching those in the reference Eylea formulation [27]. The charge variant profile, largely determined by the glycosylation patterns of aflibercept, is a critical attribute that must align closely with the original product. Although IEF fractionation is widely used to quickly assess charge variant profiles during biosimilar manufacturing, it does not provide qualitative information on the glycosylation status at the five glycosylation sites on aflibercept molecules. This study aimed to demonstrate the capability of LC-MS/MS in evaluating site-specific, sialylation-mediated glycosylation variations and to assess whether these results correlate with IEF fractionation outcomes, underscoring LC-MS/MS as a robust tool for biosimilar assessment.

## 2. Results and Discussion

### 2.1. IEF Fractionation

**Figure 1:**
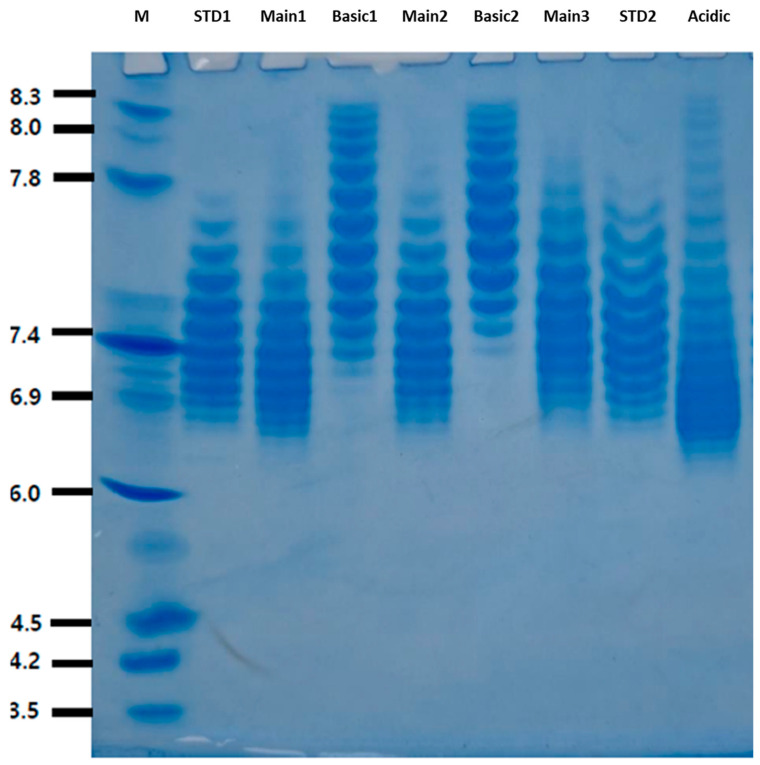
Isoelectric focusing (IEF) fractions of the VEGFR-IgG fusion protein. The gel shows the separation of protein charge variants in the pH range of 3.5 to 8.3. Lane M represents the molecular weight marker with pI values indicated on the left. Lanes labeled standard sample 1 (STD 1) and standard sample 2 (STD 2) correspond to standard samples. Lanes labeled Main1, Main2, and Main3 represent the main protein samples. Basic1 and Basic2 correspond to the basic protein variants, while the acidic lane represents the acidic protein variant. The observed band patterns indicate the distribution of charge variants across the different samples, with distinct differences in the band intensities and positions reflecting the variations in protein isoelectric points (pI) among the main, basic, and acidic samples.

Figure 1 shows the IEF result for the basic, main, and acidic fractions of the fusion protein VEGFR-IgG. The samples were separated using a Q Ceramic HyperD^®^ F column (Sartorius AG, Göttingen, Germany), which was equilibrated and washed with 20 mM Tris-HCl at pH 7.5. Elution was performed with a gradient of 0–500 mM NaCl in 20 mM Tris-HCl (pH 7.5), allowing fractionation into the main, acidic, and basic charge variants based on their isoelectric points. The leftmost lane is labeled “M”, which is the molecular weight marker. It provides reference bands of known molecular weights. The second lane from the left is a standard sample (STD), Eylea. The next three lanes are labeled “Main”, “Basic”, and “Acidic” for the IEF fractions of the fusion protein VEGFR-IgG, respectively. The main sample was analyzed in triplicate, while the basic and STD samples were analyzed in duplicate. These labels indicate the different conditions or treatments applied to the samples. The main sample produced results almost identical to the standard sample, but different IEF patterns were observed in the basic and acidic fractions. In the case of the basic fraction, since elution was performed under basic conditions, primarily basic glycans were identified. Conversely, under acidic conditions, the acidic fraction was expected to identify relatively acidic glycans.

### 2.2. Identification and Quantification of Site-Specific N-Glycopeptides

**Figure 2:**
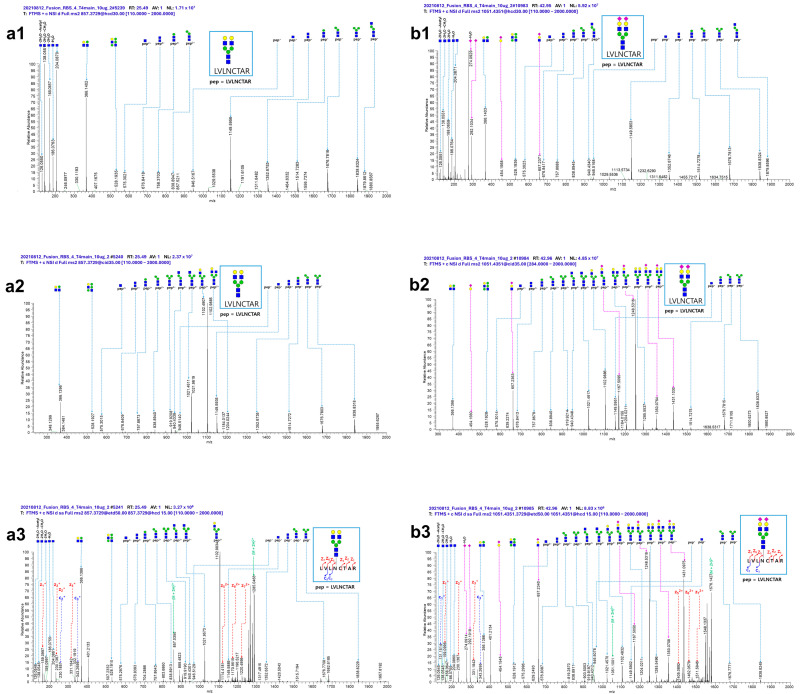
(**a1**) and (**b1**) HCD, (**a2**) and (**b2**) CID, and (**a3**) and (**b3**) EThcD spectra for the LVLNCTAR_5_4_0_0_0 (non-sialylated) and LVLNCTAR_5_4_0_2_0 (sialylated) N-glycopeptides identified in the main sample. The italic underlined N letter is the N-glycosylation site. These glycopeptides are composed of N-Acetylglucosamine (GlcNAc, blue), Mannose (Man, green), Galactose (Gal, yellow), and Sialic acid (NeuAc, purple).

In a previous study, we successfully applied the analytical techniques used here to evaluate the equivalency of Aflibercept samples from Korea, the United States, and Europe [28]. In the current study, we aimed to investigate what changes in N-glycosylation were present between the IEF fractions. Table 1 summarizes the number of N-glycopeptides identified in each sample using LC-MS/MS, and Table 2 summarizes the number of N-glycopeptides with five different types of N-glycoforms identified in three samples. We identified a total of 125 site-specific N-glycopeptides in three samples (main, basic, and acidic). The identification of N-glycopeptides was based on the MS/MS fragment ions with a false discovery rate (FDR) of less than 1%. Figure 2 shows the (a)-1 higher-energy collisional dissociation (HCD), (a)-2 collision-induced dissociation (CID), and (a)-3 electron transfer higher-energy collision dissociation (EThcD) spectra for the N-glycopeptides LVLNCTAR_5_4_0_0_0 (non-sialylated) and LVLNCTAR_5_4_0_2_0 (sialylated). Although the LVLNCTAR_5_4_0_0_0 (non-sialylated) and LVLNCTAR_5_4_0_2_0 (sialylated) N-glycopeptides exhibited different sensitivities in the MS analysis, both non-sialylated and sialylated N-glycopeptides were well-characterized by I-GPA. The HCD, CID, and EThcD spectra for LVLNCTAR_5_4_0_0_0 (non-sialylated) and LVLNCTAR_5_4_0_2_0 (sialylated) were identified by the oxonium ions from HCD, glycan-cleaved glycopeptide fragment ions (B/Y) from HCD and CID, and peptide fragment ions (c/z) from EThcD. The (a)-1–(a)-3 spectra in Figure 2 do not contain NeuAc-specific oxonium ions; however, the (b)-1–(b)-3 spectra do, showing ions at *m*/*z* 274.0926 (NeuAc-H2O), 292.1031 (NeuAc), 454.1559 (Hex1NeuAc1), and 657.2365 (Hex1GlcNAc1NeuAc1). Specifically, glycan-cleaved glycopeptide fragment ions related to the sialylated glycan, such as m/z 1167.5095 (Hex3GlcNAc3NeuAc1, 2+), 1248.5316 (Hex4GlcNAc3NeuAc1, 2+), 1350.0764 (Hex4GlcNAc4NeuAc1, 2+), and 1431.1030 (Hex5GlcNAc4NeuAc1, 2+), as well as peptide fragment ions containing the Hex5GlcNAc4Fuc0NeuAc2 glycan (*m*/*z* 1405.0892 (z5, 2+), 1462.0579 (z6, 2+), and 1511.5946 (z7, 2+)), were detected only in the sialylated LVLNCTAR_5_4_0_2_0 glycopeptide.

A total of 43, 47, and 39 N-glycopeptides were quantified in the main, basic, and acidic samples, respectively. In the main sample, out of a total of 43 N-glycopeptides, 6 N-glycopeptides were quantified from VEGFR-1, 29 from VEGFR-2, and 8 from IgG. It was observed that VEGFR-2 exhibited a relatively higher abundance of N-glycosylation. All six N-glycopeptides from VEGFR-1 were fucosylated, and four out of the six were sialylated. In VEGFR-2, 18 were sialylated, and 5 high-mannose types were quantified. Finally, in IgG, four out of eight N-glycopeptides were sialylated. Similarly, in the basic sample, 5 N-glycopeptides were quantified from VEGFR-1, 36 from VEGFR-2, and 6 from IgG, confirming that VEGFR-2 had a higher abundance of N-glycosylation. Lastly, in the acidic sample, 5 N-glycopeptides were quantified from VEGFR-1, 32 from VEGFR-2, and 2 from IgG. Detailed information on the quantified N-glycopeptides for the main, basic, and acidic samples is provided in Supplementary Tables S1, S2, and S3, respectively. The types of N-glycosylation for the quantified N-glycopeptides are also summarized.

### 2.3. The Relative Abundance of N-Glycosylation in IEF Fraction Samples

**Figure 3:**
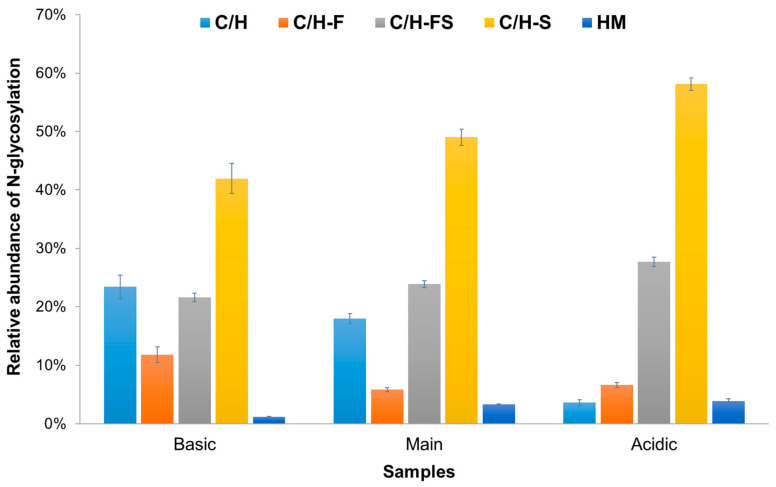
The relative abundance of N-glycosylation across three different samples: basic, main, and acidic. The y-axis represents the relative abundance of N-glycosylation, which is scaled from 0 to 100 percent. Each bar in the figure is divided into different colored segments, representing various types of N-glycosylation: C/H (complex/hybrid type), C/H-F (complex/hybrid type with fucose), C/H-FS (complex/hybrid type with fucose and sialic acid), C/H-S (complex/hybrid type with sialic acid), and HM (high-mannose type).

Figure 3 shows a bar chart that shows the relative abundance of N-glycosylation across the three different sample types (basic, main, and acidic). Figure 3 shows that C/H-S was the most abundant type in all samples, with percentages of 41.9%, 49.1%, and 58.2%, respectively. The HM type had the least relative abundance in each sample (1.2%, 3.3%, and 3.9%, respectively). Overall, although the number of quantified N-glycopeptides was similar, as shown in Table 2, it was confirmed that N-glycoforms containing sialic acid were most abundant in the acidic sample and least abundant in the basic sample. The correlation between the results of IEF and the presence of sialic acid was verified through LC-MS/MS analysis. The N-glycopeptide with only fucose attached (C/H-F) was identified in 23 N-glycopeptides in the basic sample, 19 in the main sample, and 7 in the acidic sample, as shown in Table 2. It was most abundant in the basic sample, at 11.8%, while similar results were observed in the main (5.8%) and acidic (6.6%) samples. The relative abundance of N-glycosylation without fucose or sialic acid (C/H) was highest in the basic sample at 23.5%, while, in contrast, it significantly decreased to 3.6% in the acidic sample.

**Figure 4:**
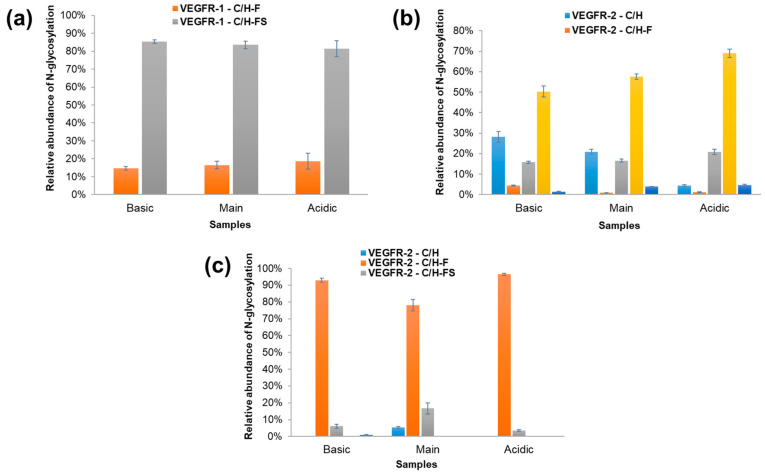
A comparison of the different N-glycoforms in the (**a**) VEGFR-1, (**b**) VEGFR-2, and (**c**) IgG domains across the three samples: basic, main, and acidic. The different glycoforms are represented by different colors in the bar chart. Blue (VEGFR-1-C/H), yellow (VEGFR-1-C/H-F), green (VEGFR-1-C/H-FS), pink (VEGFR-1-C/H-S), and purple (VEGFR-1-HM).

Figure 4 shows the results of examining the differences in N-glycoforms separately for VEGFR-1 (Figure 4a), VEGFR-2 (Figure 4b), and the IgG domain (Figure 4c). These figures provide a comparative view of the distribution of various glycopeptide types in the three different samples, highlighting differences in their relative abundances. Figure 4a indicates that the C/H-FS glycoform of VEGFR-1 was the most prevalent across all the samples, with relative abundances of 85.3%, 83.5%, and 82.0% in the basic, main, and acidic samples, respectively. In contrast, the C/H-F glycoform constituted the remaining portion, accounting for 14.7% in the basic sample, 16.5% in the main sample, and 18.0% in the acidic sample. Other glycoforms, such as C/H, C/H-S, and HM, were not observed in the VEGFR-1 region.

Figure 4b shows a comparison of the different N-glycoforms in the VEGFR-2 region across the three samples. In contrast with VEGFR1, all five representative N-glycoforms were detected in the VEGFR2 region. Figure 4b indicates that the C/H-S glycoform was the most prevalent in all samples (50.3%, 57.7%, and 69.1%, respectively). It was confirmed that the acidic sample showed the highest relative abundance. In contrast, the relative abundance of C/H significantly decreased in the acidic sample (4.3%) compared with the basic sample (28.2%). Similar to VEGFR1, C/H-FS glycoforms also accounted for a significant proportion in VEGFR2, and a slight increase was observed in the acidic sample (15.8%, 16.5%, and 20.8% in the basic, main, and acidic samples, respectively). In the VEGFR2 region, the N-glycoform containing only fucose and HM was present in the smallest amounts. C/H-F was most abundant in the basic sample, while HM was found to be highest in the acidic sample.

Figure 4c shows a detailed comparison of the different N-glycoforms present in the IgG domain across the three samples. Figure 4c indicates that the C/H-F glycoform was the most prevalent across all samples, with relative abundances of 93.0% in the basic sample, 78.2% in the main sample, and 96.5% in the acidic sample. Furthermore, the analysis within the IgG region reveals that the N-glycoform containing both fucose and sialic acid (C/H-FS) was relatively most abundant in the main sample, accounting for 16.6% of the glycoforms in this sample. This indicates a distinct glycosylation profile in the main sample compared with the basic and acidic samples, where the presence of sialic acid in conjunction with fucose was more pronounced. This finding underscores the variability in glycosylation patterns across the different sample conditions, with the main sample showing a unique glycoform distribution in the IgG region. The C/H glycoform was exclusively observed in the main sample, indicating a specific glycosylation pattern in this condition, while the HM glycoform was only detected in the basic sample, albeit at a very low abundance of 0.9%. Notably, the C/H-S glycoform was absent across all samples, suggesting that this particular glycoform was either not produced or was present at levels below the detection threshold in the IgG region.

In this study, we observed that the differences in the sialic acid content between the basic, main, and acidic samples were significant in the VEGFR-2 region, while no notable differences were found in the VEGFR-1 and IgG regions. Sialic acids, as negatively charged residues, play a critical role in stabilizing receptor interactions and modulating a protein’s bioavailability and activity. The elevated sialic acid content observed in the acidic fraction of VEGFR-2 likely reflects its functional importance in enhancing VEGF binding and signaling efficiency. Meanwhile, the relatively stable sialic acid content in the VEGFR-1 and IgG regions indicates that these domains are more structurally conserved and less reliant on dynamic glycosylation changes for their functions. Variations in sialylation across VEGFR-IgG domains can significantly affect the pharmacokinetics and stability of a protein. Sialylation levels play a critical role in determining a protein’s half-life and immunogenicity [29]. Insufficient sialylation may result in increased in vivo reactivity, potentially reducing therapeutic efficacy. Consequently, differences in sialylation between the VEGFR-1, VEGFR-2, and IgG domains could impact binding efficiency, bioavailability, and overall stability, emphasizing the need for targeted optimization for therapeutic applications. CHO-K1 cells, commonly used in biopharmaceutical production, are optimal for producing the VEGFR-IgG fusion protein due to their stability, adaptability to various culture conditions, and capacity for complex post-translational modifications like glycosylation, ensuring therapeutic relevance and consistent quality.

**Figure 5:**
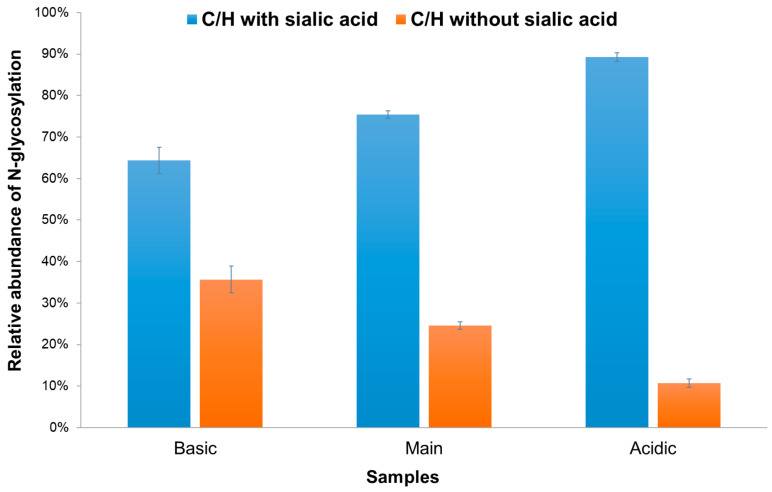
The relative abundance of N-glycopeptides with and without sialic acid of the complex type across the three different samples.

The relative abundance of N-glycopeptides with and without sialic acid of the complex type across the three different samples is shown in Figure 5. In the basic sample, it was found that 63.5% of the total contained sialic acid. This proportion was observed to gradually increase in the main (72.9%) and acidic (85.9%) samples. As a result, the LC-MS/MS analysis confirmed that glycoforms containing sialic acid were predominantly present in the acidic sample, which was collected from the acidic charge variant fraction.

The LC-MS/MS-based site-specific sialylation analysis method developed in this study holds significant potential for industrial applications in the development and quality control of biopharmaceuticals. By monitoring sialylation levels, this technique enables reliable quality control for biosimilar products, allowing manufacturers to predict the impact of glycosylation patterns on therapeutic efficacy and stability. This approach may play a crucial role in optimizing the development and production processes for customized biotherapeutics.

## 3. Materials and Methods

### 3.1. Materials and Reagents

Amicon^®^ Ultra centrifugal filters were sourced from Merck Millipore in Burlington, MA, USA. The C18 trap column was obtained from Harvard Apparatus located in Holliston, MA, USA. For protein digestion, trypsin was purchased from Promega in Madison, WI, USA. Reduction and alkylation reagents, including phosphate-buffered saline (PBS), 1,4-dithiothreitol (DTT), and iodoacetamide (IAA), were acquired from Sigma Aldrich in St. Louis, MO, USA. Additionally, trifluoroacetic acid (TFA), ammonium bicarbonate (ABC), and formic acid (FA) were also obtained from Sigma Aldrich in St. Louis, MO, USA. High-purity water and acetonitrile (ACN) (MS-grade) were procured from Merck Millipore in Darmstadt, Germany.

### 3.2. Preparation of VEGFR-IgG Fusion Protein Fractions

The CHO-k1 cell line was purchased from ATCC (catalog number: CCL-61), expanded first in F-12K (ATCC 30-2004) medium containing 10% fetal bovine serum (ATCC 30-2020) in static culture and then adapted to CDM4CHO (Hyclone SH30558.02), which is a serum-free chemically defined medium, in suspension culture. The cell lines for the VEGFR-IgG fusion protein, generated using suspension-adapted CHO-k1 cells in a chemically defined medium, were transfected by electroporation with 20 µg of the expression vector by a recombination-based transfection protocol. Recombinant aflibercept protein was produced in suspension-adapted CHO cells stably transfected with an aflibercept expression vector controlled by a CMV promoter and incorporating a puromycin-resistant gene as a selective marker. Transfection was conducted using conventional electroporation, and stably expressing cell lines were selected through puromycin screening [30]. The transfected cells were subjected to medium changes and antibiotic selection with puromycin dihydrochloride (10 µg/mL) every 48 h for 10–15 days. The growth and specific productivity rates (SPRs) of the stably high-expressing transfected cells, selected by a MoFlo-XDP (Beckman Coulter, Indianapolis, IN, USA), were monitored by a Clone Select Imager (CSI, Molecular Devices, San Jose, CA, USA) and by ELISA assay [30]. The cell viability (≥90%), growth rates, and specific productivity rate (SPR) were monitored using the Clone Select Imager and ELISA, with set thresholds for consistent proliferation and protein production. The transfection efficiency was confirmed post-electroporation, and antibiotic resistance was assessed by culturing cells in 10 µg/mL of puromycin for 10–15 days. The long-term stability of expression in the MoFlo-XDP-selected clones was tracked to ensure performance consistency over multiple passages.

Fed-batch for the final top three high-expressing clones was performed by seeding 1.5 × 10^7^ cells in 30 mL of growth medium and 10 µg/mL of puromycin. The cells were supplemented with a feeding medium every 48 h, maintained at 37 °C for 10–12 days, and harvested. The VEGFR-IgG fusion protein was purified from the cell culture supernatant using HiTrap Mabselect SuRe protein A columns, and the resulting protein was subjected to PBS buffer exchange, desalting, and concentration using Amicon^®^ Ultra centrifugal filters (Merck Millipore, Burlington, MA, USA), and IEF fractionation was conducted [28]. The supernatant was loaded onto the filters and centrifuged at 4000× *g* for 30 min at 4 °C to concentrate the protein. Buffer exchange was performed by adding PBS and repeating the centrifugation process 3 times. The concentrated protein was collected, and the final concentration was measured using a BCA assay. The purified samples were stored at 4 °C or −80 °C for further analysis.

### 3.3. Sample Analysis with LC-MS/MS

The LC-MS/MS analysis method in this study was conducted based on our previous work [28]. For analysis, 100 µg of the protein sample prepared by IEF fractionation was denatured with ABC (50 mM) and urea (2 M) and reduced by DTT (100 mM) for 1 h at 35 °C. After incubation, the sample was alkylated with IAA (100 mM) for 1 h in a darkroom and digested with trypsin (0.125 µg/µL) overnight at 37 °C. The digested samples were reconstituted in deionized water (DW) with 0.1% formic acid (FA) and analyzed using an LC-MS/MS system equipped with an Easy nLC 1200 (Thermo Fisher Scientific, Waltham, MA, USA) and an Orbitrap Fusion Lumos mass spectrometer (Thermo Fisher Scientific), which included a nanoelectrospray ion (ESI) source (EASY-Spray Sources, Thermo Fisher Scientific). The peptides were trapped and separated using a 75 μm × 2 cm C18 pre-column (nanoViper, Acclaim Pep-MapTM100, Thermo Fisher Scientific) and an analytical C18 column (75 μm × 50 cm Pep-MapTM RSLC, Thermo Fisher Scientific), respectively, at a flow rate of 250 nL/min. The mobile phases were water with 0.1% FA (phase A) and 80% acetonitrile with 0.1% FA (phase B). The LC gradient involved ramping from 1.6% B to 4.8% B over 1 min, increasing to 8% B over 12 min, to 28% B over 60 min, and to 80% B over 1 min. This was followed by maintaining 80% B for 5 min and decreasing to 1.6% B over 3 min. The analytical column was re-equilibrated with 1.6% B for 8 min before the next run. The electrospray voltage was set to approximately 1900 V. During the chromatographic separation, the Orbitrap Fusion Lumos operated in data-dependent mode with MS1 and MS2 automatically switching within a 3 s cycle time. The full-scan MS1 range was set from 400 to 2500 *m*/*z*, acquired by the Orbitrap with a maximum ion injection time of 100 ms at a resolution of 120,000 and an automatic gain control (AGC) target value of 1.0 × 10^6^. For the MS2 spectra, high-energy collision dissociation (HCD) was performed in the ion routing multipole, and collision-induced dissociation (CID) was performed in the linear ion trap. The product of fragmentation was then delivered to the Orbitrap analyzer at a resolution of 30,000, using a 35% and 30% normalized collision energy, respectively, with an AGC target value of 1.0 × 10^5^ and a maximum ion injection time of 150 ms. Previously fragmented ions were excluded for 30 s with a 10 ppm mass tolerance. Internal calibration was conducted using the mass peak at 445.12003 *m*/*z*, released from polysiloxane from the silica capillary of the NanoSprayer (Thermo Fisher Scientific, Waltham, MA, USA).

### 3.4. Data Analysis

An Integrated GlycoProteome Analyzer (I-GPA) was employed to automatically identify N-glycosite-specific glycopeptides from the VEGFR-IgG fusion proteins [31]. Initially, N-glycopeptide tandem spectra were selected based on 15 specific oxonium ions of N-glycans. Candidate N-glycopeptides were then identified by comparing their experimental MS isotope patterns with theoretical patterns from the GPA database (GPA-DB). This database includes possible tryptic peptides containing N-glycosites of VEGFR-IgG proteins, combined with 351 N-glycans (331 retrosynthetic glycans and 20 polylactosamine series glycans, such as penta- and hexa-saccharides).

The identification of N-glycopeptides was based on their Y-scores, which quantifies the match between experimental and theoretical fragment ions. From the HCD and CID MS/MS spectra, the B/Y ions (resulting from the glycosidic bond cleavage of N-glycans at N-glycosylation sites) and b/y ions (resulting from peptide bond cleavage) were used for Y-scoring. The I-GPA search parameters included fixed carbamidomethyl cysteine modification and mammalian N-glycans for N-sites, allowing for one missed tryptic cleavage. The mass tolerances for precursor and fragment ions (HCD, CID, and ETD) were set at 0.02 Da. N-glycopeptides were identified if their Y-score was within 0.01 of the false discovery rate (FDR) threshold. Label-free quantification of N-glycopeptides was performed using 3 replicate LC-MS/MS measurements. The quantitative values of the identified N-glycoproteins from each sample were normalized for comparative analysis. N-glycopeptides with coefficient of variation (CV, %) values of less than 30% were then quantified.

In this study, N-glycopeptides were represented by the peptide amino acid sequence and the counts of hexoses (Hex), N-acetylglucosamines (GlcNAc), fucoses (Fuc), N-acetylneuraminic acids (NeuAc), and N-glycolylneuraminic acids (NeuGc). For example, the N-glycopeptide EEQYNSTYR_4_4_1_0_0 included the peptide sequence EEQYNSTYR, 4 Hex, 4 GlcNAc, 1 Fuc, 0 NeuAc, and 0 NeuGc.

## 4. Conclusions

This study effectively demonstrated the application of the site-specific N-glycosylation analysis of the Fc-fusion protein to evaluate sialylation levels at individual glycosylation sites, revealing a clear correlation with the observed variations across IEF fractions. These findings are instrumental for optimizing Aflibercept production processes and improving its therapeutic efficacy, contributing to enhanced treatment outcomes. The use of LC-MS/MS, with its ability to deliver precise, detailed site-specific glycosylation profiles, highlights its indispensable role in maintaining the consistency and quality of biosimilars. Furthermore, the method’s capability to detect subtle glycosylation variations, such as sialylation differences across charge variants, underscores the significance of advanced mass spectrometry in the comprehensive development, regulation, and quality assurance of biotherapeutics. By facilitating a deeper understanding of glycosylation patterns, this research supports the advancement of biopharmaceuticals to meet stringent clinical standards, ultimately broadening the potential for more effective and tailored medical treatments.

## Figures and Tables

**Table 1:** The number of N-glycopeptides identified in the samples.

Sample	The Number of Identified N-Glycopeptides	The Number of Identified N-Glycopeptides with Sialic Acid	The Number of Quantified N-Glycopeptides	The Number of Quantified N-Glycopeptides with Sialic Acid
Basic	103	52	47	23
Main	104	57	43	26
Acidic	73	47	39	25

**Table 2:** The number of N-glycopeptides with five different types of glycoforms identified in the samples.

Sample	C/H	C/H-F	C/H-FS	C/H-S	HM
Basic	14	23	29	23	11
Main	14	19	31	26	12
Acidic	10	7	26	21	8

Types of N-glycosylation: C/H (complex/hybrid type), C/H-F (complex/hybrid type with fucose), C/H-FS (complex/hybrid type with fucose and sialic acid), C/H-S (complex/hybrid type with sialic acid), and HM (high-mannose type).

## Data Availability

The original contributions presented in the study are included in the article/Supplementary Materials, further inquiries can be directed to the corresponding author.

## Supplementary Materials

The following supporting information can be downloaded at: https://www.mdpi.com/article/10.3390/molecules29225393/s1, Table S1: List of N-glycopeptides quantified in the Main sample, along with their normalized peak areas and N-glycan types, Table S2: List of N-glycopeptides quantified in the Basic sample, along with their normalized peak areas and N-glycan types, Table S3: List of N-glycopeptides quantified in the Acidic sample, along with their normalized peak areas and N-glycan types.

## Abbreviations

LC: liquid chromatography; MS, mass spectrometry; CID, collision-induced dissociation; CV, coefficient of variation; Hex, hexose; HexNAc, *N*-acetylhexosamine; NeuAc, *N*-acetylneuraminic acid; Fuc, fucose.
